# Exploring Ganweikang Tablet as a Candidate Drug for NAFLD Through Network Pharmacology Analysis and Experimental Validation

**DOI:** 10.3389/fphar.2022.893336

**Published:** 2022-06-14

**Authors:** Chuanrui Ma, Xinyu Wang, Jing Zhang, Yun Zhao, Yunqing Hua, Chao Zhang, Guobin Zheng, Guangyan Yang, Jianli Guan, Huahuan Li, Meng Li, Lin Kang, Jiaqing Xiang, Guanwei Fan, Shu Yang

**Affiliations:** ^1^ First Teaching Hospital of Tianjin University of Traditional Chinese Medicine, Tianjin, China; ^2^ National Clinical Research Center for Chinese Medicine Acupuncture and Moxibustion, Tianjin, China; ^3^ State Key Laboratory of Component-based Chinese Medicine, Tianjin University of Traditional Chinese Medicine, Tianjin, China; ^4^ Department of Geriatrics, Shenzhen People’s Hospital (The Second Clinical Medical College, Jinan University, The First Affiliated Hospital, Southern University of Science and Technology), Shenzhen, China; ^5^ NHC Key Laboratory of Hormones and Development, Tianjin Key Laboratory of Metabolic Diseases, Chu Hsien-I Memorial Hospital and Tianjin Institute of Endocrinology, Tianjin Medical University, Tianjin, China; ^6^ Henan Fusen Pharmaceutical Co., Ltd., Henan, China; ^7^ The Biobank of National Innovation Center for Advanced Medical Devices, Shenzhen People’s Hospital, Shenzhen, China; ^8^ Integrated Chinese and Western Medicine Postdoctoral Research Station, Jinan University, Guangzhou, China

**Keywords:** network pharmacology, NAFL, hepatic steatosis, NASH, inflammation

## Abstract

Nonalcoholic fatty liver disease (NAFLD) is defined as liver disease in which more than 5% of hepatocytes are steatotic with little or no alcohol consumption. NAFLD includes benign nonalcoholic fatty liver (NAFL) and nonalcoholic steatohepatitis (NASH). Importantly, NASH is an advanced progression of NAFL and is characterized by steatosis, hepatocyte ballooning, lobular inflammation, and fibrosis. However, to date, no drugs specifically targeting NAFLD have been approved by the FDA. Therefore, a new drug or strategy for NAFLD treatment is necessary. However, the pathogenesis of NAFLD is complex and no single-target drugs have achieved the desired results. Noticeably, traditional Chinese medicine formulations are a complex system with multiple components, multiple targets, and synergistic effects between components. The Ganweikang tablet is a compound formula based on traditional Chinese medicine theory and clinical experience. In this study, network pharmacology analysis indicates Ganweikang tablet as a candidate for NAFLD treatment. Furthermore, we evaluated the therapeutic effects of Ganweikang tablet on the NAFL and NASH and tried to clarify the underlying molecular mechanisms in animal models and cell experiments. As expected, Ganweikang tablet was found to improve NAFL and NASH by modulating inflammation, apoptosis, and fatty acid oxidation by inhibiting NFκB, caspase-8, and activating PPARα, which not only indicates that Ganweikang tablet as a drug candidate but also provides a theoretical basis of Ganweikang tablet for the treatment of NAFL and NASH.

## Introduction

Nonalcoholic fatty liver disease (NAFLD) is defined as liver disease in which more than 5% of hepatocytes are steatotic under the condition of little or no alcohol consumption (Sanyal et al., 2011). NAFLD commences with nonalcoholic fatty liver (NAFL) which is characterized by steatosis with minimal or no lobular inflammation. Throughout the disease, the accumulation of triacylglycerols leads to a fatty infiltration with inflammation, which evolves into a more severe stage of NAFLD, nonalcoholic steatohepatitis (NASH), characterized by ballooning hepatocyte degeneration, diffuse lobular inflammation, and fibrosis (Leoni et al., 2018). As NASH develops, it can progress further to cirrhosis and hepatocellular carcinoma (Goldner and Lavine, 2020). However, to date, no drugs for NAFLD have been approved by the FDA (Sanyal et al., 2015).

The mechanisms underlying NAFLD are mainly related to lipid-induced apoptosis and inflammation (Buzzetti et al., 2016). When the fatty acid catabolism is not sufficient to offset hepatic lipid overload, toxic fatty acid derivatives are generated, which lead to activating inflammatory vesicles, increased endoplasmic reticulum, oxidative stress, and hepatocyte death, thereby promoting the progression of steatosis to NASH (Koliaki et al., 2015). Furthermore, signals from stressed or damaged hepatocytes and activated macrophages drive resident hepatic stellate cells to activate myofibroblasts and produce excessive matrix proteins, which contribute to NASH to a severe stage (Friedman et al., 2018). Dysregulation of NF-κB activation has a significant effect on the development of hepatic steatosis and inflammation, and inhibition of NF-κB ameliorates inflammatory infiltration in the liver of NASH mice (Romics et al., 2004; Locatelli et al., 2013; Rom et al., 2020). In addition, the apoptotic markers, such as caspase-3 and caspase-8, were found to be significantly upregulated in NASH and NAFL mice; and inhibition of either can reduce apoptosis and inflammatory infiltration, which alleviates liver damage in NASH and NAFL mice (Hatting et al., 2013; Wang et al., 2017; Li et al., 2018; El-Derany and El-Demerdash, 2020). Therefore, targeting lipid metabolism and inflammation is an important strategy for NAFLD treatment. The current evidence suggests that pioglitazone, a PPARγ agonist that can improve insulin sensitivity, transaminases, steatosis, inflammation, and ballooning in patients with NASH and T2DM, showed the potential for treating NAFLD (Cusi, 2016; Rinella and Sanyal, 2016; Francque et al., 2021). However, the side effects such as heart failure, fracture, and weight gain caused by pioglitazone limit its further clinical application in the treatment of NAFLD (Morán-Salvador et al., 2011). More importantly, the pathogenesis of NAFLD is complex and no single-target drugs have achieved the desired results (Friedman et al., 2018). Noticeably, traditional Chinese medicine formulation is a complex system with multiple components, multiple targets, and synergistic effects between components, which is supported by the approach of network pharmacology (Hopkins, 2007; Ma et al., 2015). Therefore, traditional Chinese medicine formulation is a potential candidate for NAFLD treatment.

Ganweikang tablet is a compound formula based on traditional Chinese medicine theory and clinical experience, which shows beneficial effects on the liver and no serious adverse reactions have been observed to date. Lianqiao (Fructus Forsythiae), Fangfeng (Radix Saposhnikoviae divaricatae), Shanyinchaihu (Gypsophila pacifica), Mabiancao (Verbena officinalis), Huoxiang (Agastache rugosus), Baishu (Rhizoma Atractylodis macrocephalae), Huangqi (Radix Astragali), and Gancao (Radix Glycyrrhizae) are the main herbal ingredients of Ganweikang tablet. Previous studies have revealed that these herbals formulating Ganweikang tablet can attenuate inflammation (Zhou et al., 2019; Lee et al., 2021), liver injury (Hu et al., 2020), and apoptosis (Fattorusso et al., 2006; Hu et al., 2014; Adesso et al., 2018; Lee et al., 2021; Zhao et al., 2021), which indicates the potential and beneficial effects of Ganweikang tablet on NAFLD. However, whether it can alleviate NAFLD, especially the NAFL and NASH, remains unclear. Therefore, in this study, we attempt to determine the protective effect of Ganweikang tablet on NAFL and NASH and disclose the underlying mechanism through an approach of network pharmacology and animal model experiment as well as cell experiment.

## Methods

### Construction of Ganweikang Tablet–Chinese Herbal–Target Genes Network

HERB (http://herb.ac.cn/), a high-throughput experimental and reference Chinese medicine database, was used for mining the target genes of herbal ingredients in Ganweikang tablet (Fang et al., 2021). Totally, 232 potential target genes of the Ganweikang tablet were predicted by HERB, and the Ganweikang herb-target genes network was constructed by Cytoscape software (Shannon et al., 2003). KEGG and WIKI enrichment analysis of Ganweikang target genes was achieved by the ENRICHR online analysis tool (maayanlab.cloud/Enrichr/) (Chen et al., 2013). The ggplot2 and ggpubr packages of R software were used for the visualization of enrichment results (Ito and Murphy, 2013; Whitehead et al., 2019), including terms, gene ratio, gene counts, and p-value.

### Regents

Rabbit anti-α-SMA (Cat No. ab179467), Col4α1 (Cat No. ab6586), CD206 (Cat No. ab64693), CD68 (Cat No. ab125212), PPARα (Cat No. ab227074), and mouse anti-CD86 (Cat No. ab220188) antibodies were purchased from Abcam (Cambridge, MA). Rabbit anti-F4/80 (Cat No. #30325), NF-κB-p65 (Cat No. #8242), and phosphorylation of NF-κB-p65 (Cat No. #3033) antibodies were purchased from Cell Signaling Technology. Rabbit anti-β−actin (Cat No. AC028) was purchased from ABclonal (Wuhan, China). Mouse AST ELISA kit (Cat No. ab263882), ALT assay kit (Cat No. ab241035), ALP assay Kit (Cat No. ab267583), gamma-glutamyl transferase (γ-GT) assay kit (Cat No. ab241029), triglyceride assay kit (Cat No. ab65336), free fatty acid assay (Cat No. ab65341), ROS detection assay (Cat No. ab139476), caspase-3 assay kit (Cat No. ab39401), and mitochondrial complex I enzyme activity microplate assay kit (Cat No. ab109721) and mitochondrial complex III activity assay kit (Cat No. ab287844) were obtained from Abcam (Cambridge, MA). Glycyrrhetinic acid (Cat No. HY-N0375), betaine (Cat No. HY-B0710), ursolic acid (Cat No. HY-N0140), and wogonin (Cat No. HY-N0400) were purchased from MedChemExpress (Shanghai, China).

### *In Vivo* Studies With Animals

All experimental protocols for animal care and *in vivo* studies conform to the Guide for the Care and Use of Laboratory Animals published by the National Institutes of Health (NIH) (NIH Publication No. 85–23, revised 1996). The animal experiments were conducted under the ARRIVE guidelines (McGrath and Lilley, 2015; Lilley et al., 2020; Percie du Sert et al., 2020) and were approved by the Ethics Committee of the Second Clinical School of Jinan University (Shenzhen People’s Hospital). Male C57BL/6J wild-type mice (8 ± 0.5 weeks old) were purchased from Gempharmatech Co., Ltd (Nanjing, Jiangsu, China). The mice were housed in the SPF unit of the Shenzhen People’s Hospital Animal Centre (12-h light cycle from 8 a.m. to 8 p.m., 23 ± 1°C, 60–70% humidity) and maintained on standard rodent food with free access to water as we previously reported (Xiang et al., 2021). A maximum of 5 mice were housed in each cage bedding with corn cobs, and the mice were given a 7-days acclimatization period before the experiment. The mice were randomly divided into 12 groups and were induced into NAFL and NASH models, respectively. In detail, male C57BL/6J mice were fed a high-fat diet (HFD: 41% fat plus 0.5% cholesterol) for 8 weeks to induce the NALF model; or an MCD diet with 0% methionine and 0% choline for 1 week to induce the NASH model. In the current study, different doses of Ganweikang tablets (low dose, L, 336 mg/kg/day; medium dose, M, 672 mg/kg/day; high dose, H, 1,344 mg/kg/day) were administered in an *in vivo* experiment to detect the therapeutic effects on NAFL and NASH. Fenofibrate (Feno, 100 mg/kg) was used as the positive control drug as previously reported (Lefere et al., 2020). In the NAFL experiment, the mice were treated with normal chow (NC), high-fat diet (HFD), HFD with fenofibrate (Feno), HFD with low-dosage Ganweikang tablet (HFD + L), HFD with medium-dosage Ganweikang tablet (HFD + M), and HFD with high-dosage Ganweikang tablet (HFD + H). In the NASH experiment, the mice were treated with normal chow (NC), MCD, MCD with fenofibrate (Feno), MCD diet with low-dosage Ganweikang tablet (MCD + L), MCD with medium-dose Ganweikang tablet (MCD + M), and MCD with high-dosage Ganweikang tablet (MCD + H). At the end of the experiment, all mice were anesthetized in a CO_2_ chamber with the blood and liver being collected. The blood was rested for 4 h and centrifuged (3,000 rpm/min) to obtain the serum that was used to test for γGT, AST, ALT, and ALP to evaluate liver function by the fully automatic biochemical analyzer.

### Liver Histology

The liver was pathologically sectioned to assess the lesion. Formalin-fixed and paraffin-embedded liver sections were assessed by hematoxylin and eosin (H&E) for liver histology, Sirius red for fibrosis, and oil red O staining for lipid droplet accumulation. The nonalcoholic fatty liver activity score (NAS) and fibrosis stage were assessed according to the NASH CRN scoring system (Kleiner and Makhlouf, 2016). Histological scoring was performed blinded to the knowledge of the assessor of the treatment received. In addition, CD68, CD206, CD86, F4/80, Col4α1, and αSMA in the liver were detected by immunofluorescence staining to assess inflammation and fibrosis.

### Quantitative Real-Time Polymerase Chain Reaction, Western Blot Assay, and ELISA Assay

The method of RNA extraction and cDNA obtained was used as we reported (Xiang et al., 2021). Briefly, qRT-PCR was performed using the ABI Step One Plus ™ Real-time PCR system (Applied Biosystem) with specific primers (Supplementary Table S1). The relative mRNA level of target genes was normalized using the level detected in the control group as 1. The expression of *IL-1*β*, TNF-*α*, IL-6, Mmp9, Bax, Ccl2, Ccr2, Cxcl1, IL-4, Tgf-β1, Col1α1, Col1α2, Timp-1, Bcl2, and Caspase3* mRNA was normalized by *β-actin* mRNA in the corresponding samples to reflect the transcript levels of inflammation, fibrosis, and apoptosis in the liver. Western blot was performed as we previously reported (Xiang et al., 2021). First, the collected tissue is lysed and homogenized in lysis buffer (Sigma-Aldrich; St. Louis, MO, United States), and total protein was obtained according to the classical protocol. Dilute fresh primary antibody (1:1,000) or anti-β-actin antibody (1:5,000) and fresh HRP-conjugated anti-rabbit or mouse IgG (1:5,000) in 1% fresh non-fat dry milk in PBS. The expression of NFκB-p65, p-NFκB-p65, caspase8, PPARα, and β-actin were determined by Western blot as we reported. The livers were cryogenically homogenized, and the TG and FFA levels in them were measured by ELISA according to the instructions.

### Molecular Docking

Discovery Studio (DS) is molecular modeling software for drug discovery and protein structure analysis (Zhang et al., 2020). In this study, the DS 2019 version was used to molecularly dock the active ingredient of the Ganweikang tablet with its target protein. The structures of the active ingredients and proteins were downloaded from the NCBI database (https://www.ncbi.nlm.nih.gov/pccompound) and the Protein Data Bank (https://www.rcsb.org), respectively (Barrett et al., 2013; Karuppasamy et al., 2020). The proteins required a series of preparations before docking, including the removal of water molecules, the addition of hydrogen atoms, and the setting up of active pockets. CDocker, an algorithm that can precisely dock any number of ligands to a single protein receptor, was used for molecular docking in this study (Wu et al., 2003).

### Cell Culture

HepG2 cells (a human hepatic cell line, were purchased from ATCC, Manassas, VA, United States) and were cultured in a complete DMEM medium (10% FBS, 50 μg mL^−1^ penicillin/streptomycin, and 2 mM glutamine). When cells grew to ∼ 90% confluence in the culture dish, the complete DMEM medium was replaced by the serum-free medium. Cells received the treatment by glycyrrhetinic acid (GA, 20 μg/ml), ursolic acid (UA, 5 μM), betaine (bet, 5.0 mg/ml), and wogonin (wog, 10 μg/ml) in the presence or absence of LPS (1 μg/ml). After treatment, RNA and protein were extracted for the following q-RT-PCR and Western blot assay.

### Data Analysis

The size of the *in vivo* study was calculated based on previous studies and pre-experiments. No outliers were identified in this study, and no data were excluded from the analysis. All data were generated from at least three independent experiments. The density of the target band and qRT-PCR target gene mRNA was normalized to β-actin in the corresponding sample to reduce variance. All values were normalized to the mean value of the experimental control group. The density of the images was quantified through ImageJ software (National Institutes of Health, Bethesda, MD, United States). The significance of differences between two or more sample means was tested by one-way ANOVA with Bonferroni correction. The declared group size is the number of independent values, and that statistical analysis was carried out using these independent values. All data are expressed as mean ± SEM. The significant difference was considered at *p* < 0.05.

### Chemical Compounds Studied in This Article

Hematoxylin (PubChem CID: 442514); eosin (PubChem CID: 11048); oil red O (PubChem CID: 62330); fenofibrate (PubChem CID: 3339); glycyrrhetinic acid (PubChem CID: 73398); betaine (PubChem CID: 247); ursolic acid (PubChem CID: 64945); and wogonin (PubChem CID: 5281703).

## Results

### Ganweikang Tablet Alleviates Hepatic Lipid Accumulation and Liver Lesion in HFD-Induced NAFL Mice

**FIGURE 1:**
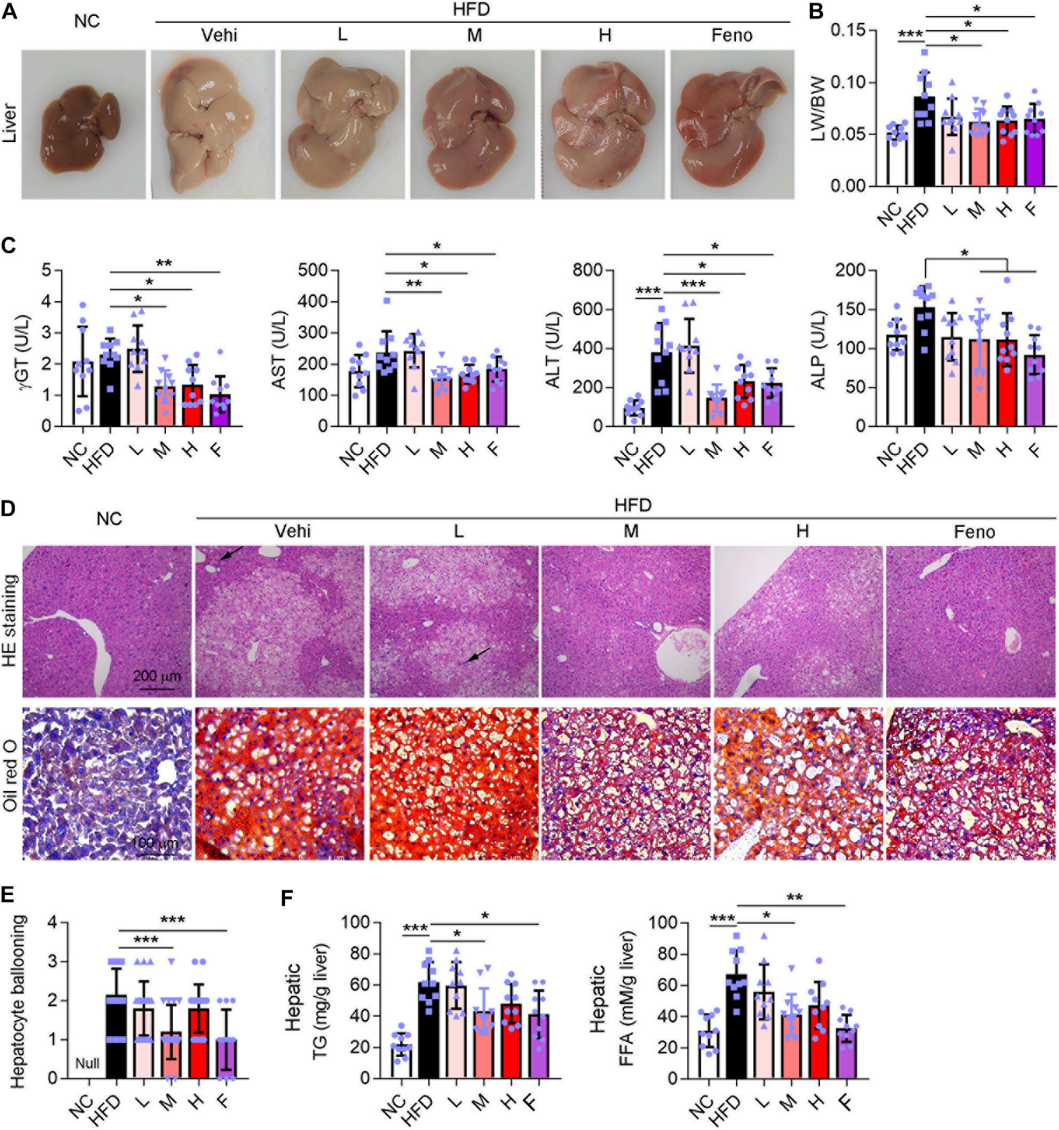
Ganweikang tablet alleviates liver lesions in HFD-induced NAFL mice. **(A)** Representative images of mouse liver. **(B)** The ratio of liver weight (LW) to body weight (BW) in mice with different treatments as indicated in the figure, *n* = 10. **(C)** Serum γGT, AST, ALT, and ALP were detected using kits, *n* = 10. **(D)** Representative images of HE staining (up panel) and oil red O staining (down panel) of mouse liver. **(E)** Hepatocyte ballooning of mice liver, *n* = 10. **(F)** Hepatic TG and FFA content were determined by kit, n = 10. *p<0.05, **p<0.01, ***p<0.001, by one-way ANOVA with Bonferroni correction.

To investigate the protective effect of the Ganweikang tablet on NAFL, C57BL/6J background mice were fed HFD for a total of 16 weeks, and different doses of the Ganweikang tablet were added to the diet at the 8th week. Mice fed HFD had whiter livers and an increased liver-to-body ratio compared to that in the NC group (Figure 1A). Compared to the mice fed only HFD, the mice given fenofibrate and a medium or high dose of Ganweikang tablets along with HFD showed a progressive reddening of the liver and a significant decrease in the liver-to-body ratio (Figures 1A, B). Similarly, liver injury-related indicators, including γGT, AST, ALT, and ALP, were significantly reduced in the fenofibrate and the medium- or high-dose Ganweikang tablet group compared to the HFD group (Figure 1C). In addition, decreased hepatocyte ballooning and lipid droplet deposition were observed in the fenofibrate group and the Ganweikang tablet group by HE and oil red O staining (Figures 1D, E). Moreover, the levels of TG and FFA were significantly lower in the liver of mice that were treated with the medium dose of Ganweikang compared to the HFD group (Figure 1F). Altogether, these data suggest that the Ganweikang tablet protects the liver from HFD-induced lipid deposition and liver injury in HFD-induced NAFL mice.

### Ganweikang Tablet Inhibits Hepatic Inflammation and Fibrosis in HFD-Induced NAFL Mice

**FIGURE 2:**
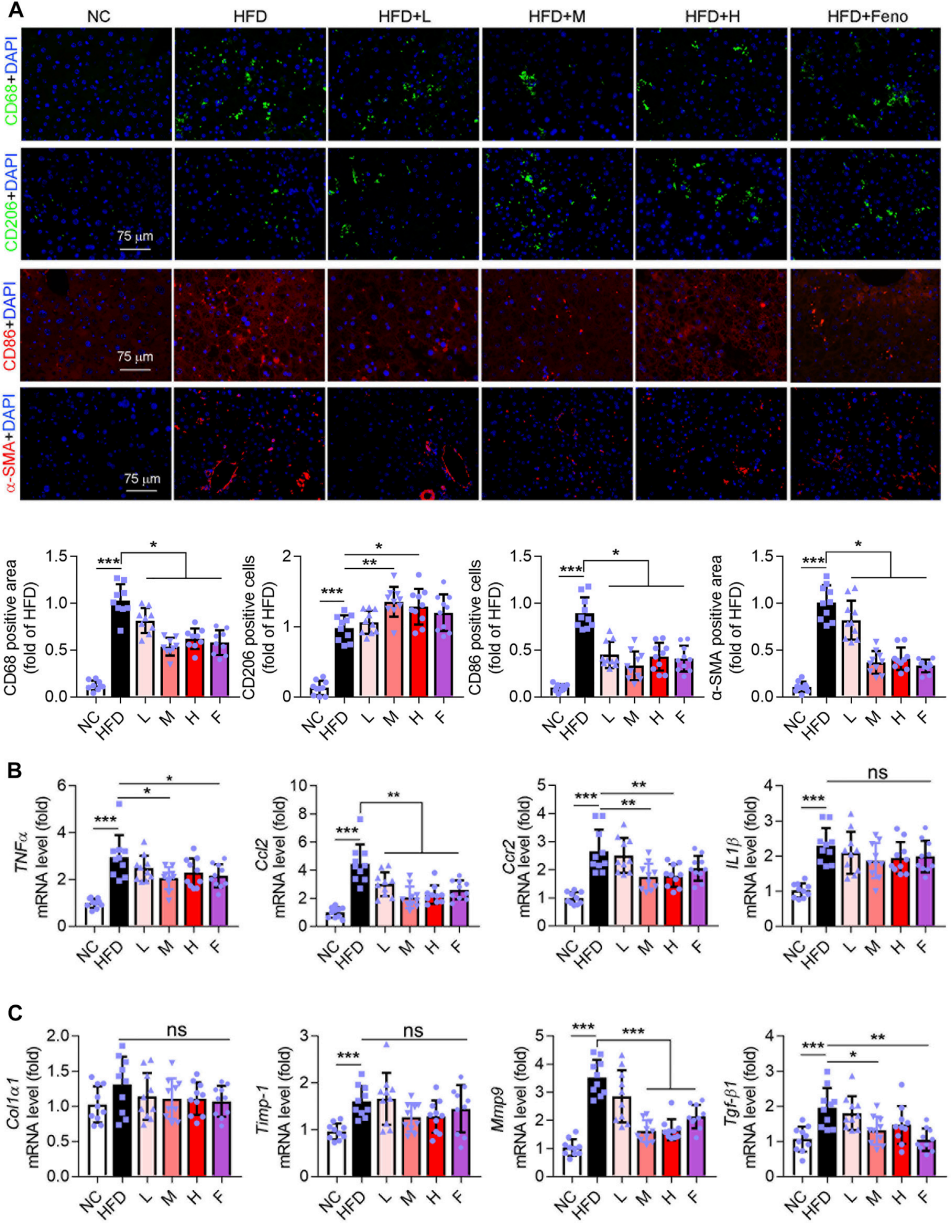
Ganweikang tablet inhibits hepatic inflammation and fibrosis in NAFL mice. **(A)** Immunofluorescence staining for CD68 (green), CD206 (green), CD86 (red), α-SMA (red), and DAPI (blue) in liver sections from NC, HFD, HFD + L, HFD + M, HFD + H, and HFD + Feno group, followed by the quantitative analysis below. **(B)** The fold changes of *TNFα*, *Ccl2*, *Ccr2*, and *IL1β* mRNA in the liver from the indicated group (*n* = 10 mice examined per group). **(C)** The mRNA fold level changes of *Col1α1*, *Timp-1*, *Mmp9*, and *Tgf-β1* in the liver from the group as indicated (n = 10 mice examined per group). *p<0.05, **p<0.01, ***p<0.001, by one-way ANOVA with Bonferroni correction. ns, no significance.

Macrophage plays an important role in liver function. CD68 is a marker for macrophages. In addition, CD86 and CD206 are the markers of macrophage M1 and M2, respectively; the M1 phenotype is associated with an inflammatory response and the M2 phenotype with an anti-inflammatory response (Wynn and Vannella, 2016; Fan et al., 2020; Dong et al., 2016). In this study, HFD intervention increased hepatic CD68^+^, CD206^+^, and CD86^+^ macrophages in mice (Figure 2A). Fenofibrate and medium-dose Ganweikang tablet administration reduced the number of CD68^+^, and CD86^+^ macrophages and increased the number of CD206^+^ macrophages (Figure 2A). Moreover, we further evaluated the expression of pro-inflammatory cytokines in the liver. Notably, similar to fenofibrate, a medium dose of Ganweikang tablet significantly inhibited the HFD-induced increase in mRNA expression of *TNFα* and *Ccl2* in the liver of mice (Figure 2B). Meanwhile, the medium- and high-dose Ganweikang tablet intervention significantly inhibited *Ccr2* expression. Compared to the HFD group, fenofibrate slightly attenuated the *Ccr2* and *IL1β* expression but without a difference (Figure 2B). In addition, Ganweikang tablet slightly reduced the *IL1β* expression but without a significant difference (Figure 2B). Hepatic inflammation and fibrosis are accompanied by NAFL development (Brunt et al., 2015). αSMA is a marker of hepatic stellate cell activation and liver fibrosis (Xiang et al., 2020). Intervention with fenofibrate or medium dose of Ganweikang tablet significantly inhibited the expression of fibrosis-related phenotype markers, including αSMA, *Mmp9*, and *Tgf-β1* (Figures 2A, C). In addition, fenofibrate and Ganweikang tablet slightly reduced the *Col1α1* and *Timp-1* expression but without significant difference (Figure 2C)*.* The aforementioned results suggest that Ganweikang tablet can reduce the inflammatory infiltration and fibrosis in the liver of HFD-fed NAFL mice, partially by which ameliorating the liver injury.

### Ganweikang Tablet Alleviates Liver Lesion in MCD-Induced NASH Mice

**FIGURE 3:**
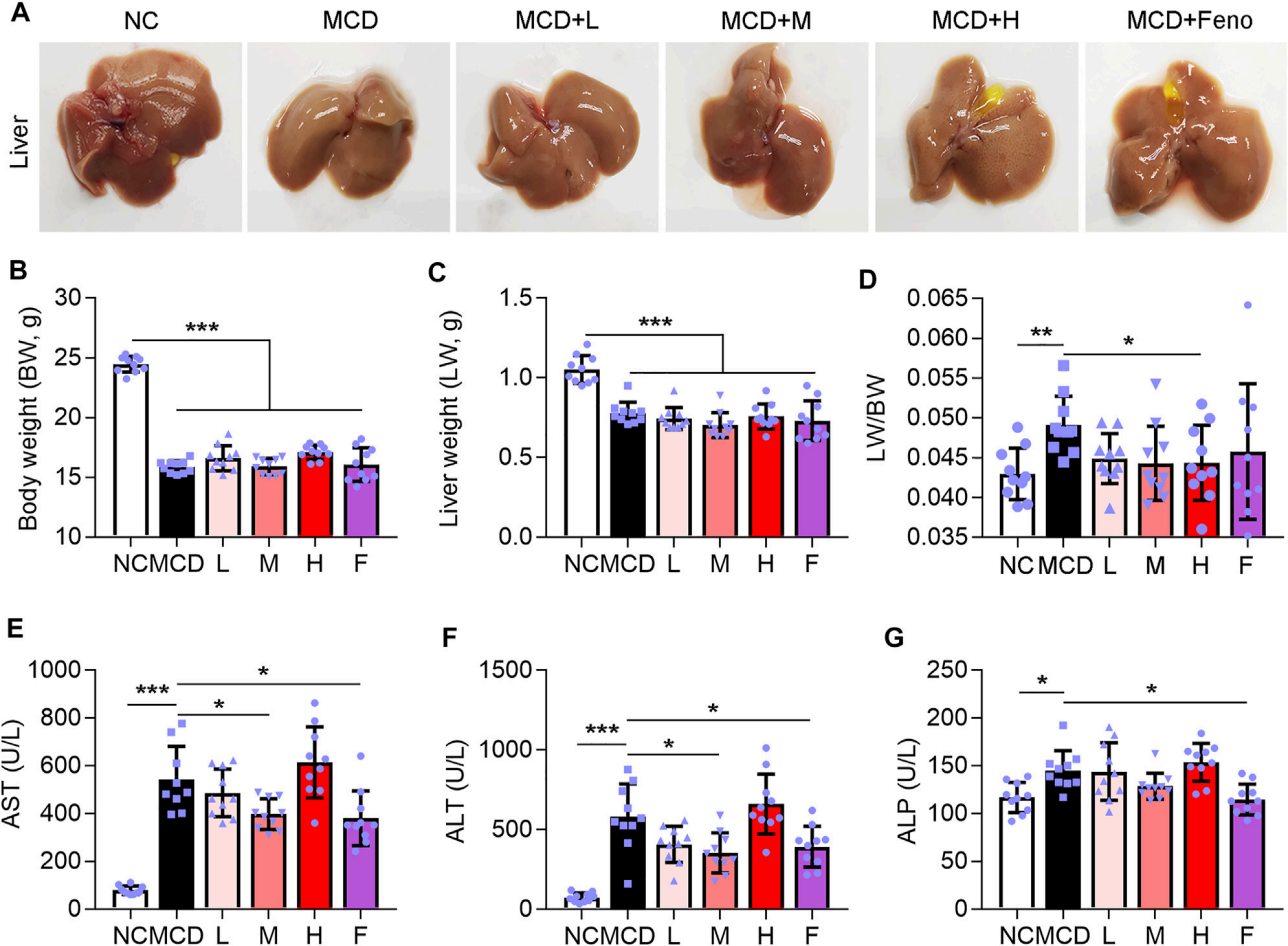
Ganweikang tablet alleviates liver lesions in MCD-induced NASH mice. Eight-week-old male mice on a C57BL/6J background were fed an MCD diet for a total of 8 weeks, with the Ganweikang tablet intervention at the fourth week, and the serum and liver were collected at the time of mouse euthanization. **(A)** Representative images of mouse liver. **(B–D)** The body weight (BW), liver weight (LW), and the ratio of LW to BW, *n* = 10 per group. **(E–G)** Serum AST, ALT, and ALP were detected by kit, *n* = 10 per group. *p<0.05, **p<0.01, ***p<0.001, by one-way ANOVA with Bonferroni correction.

NASH is a further stage of NAFL. Therefore, we further experimented to evaluate the effect of the Ganweikang tablet on NASH. Eight-week-old male mice on a C57BL/6J background were fed an MCD diet for a total of 8 weeks (NASH mice), with the fenofibrate or Ganweikang tablet intervention at the fourth week, and the serum and liver were collected at the time of mouse euthanasia. Compared to the NC group, the MCD-fed mice led to a change in the liver to whiter (Figure 3A), a decrease in body weight and liver weight (Figures 3B, C), and an increase in the liver to body ratio (Figure 3D), which indicated that the NASH model was successfully induced. The NASH model is accompanied by the development of lipotoxicity and extensive inflammatory infiltration as well as apoptosis in the liver. Despite no significant increase in the liver and body weight after treatment by Ganweikang tablet or fenofibrate compared to the MCD group (Figures 3B, C), the features of the liver in MCD-induced NASH mice, including the whiter color and the decreased liver-to-body weight ratio, were improved by the medium-dosage Ganweikang tablet or fenofibrate administration (Figures 3A, D), suggesting that Ganweikang tablet administration plays a beneficial role in the NASH treatment. In addition, we detected the liver injury-related indicators ALT and AST and observed that both were significantly elevated in MCD-induced NASH mice, fortunately, which was partially reversed by fenofibrate or medium dose of Ganweikang tablet; but no differences were found in the low or high dose administration groups (Figures 3E, F). However, the other indicator of liver damage, ALP, was decreased only in the fenofibrate group (Figure 3G). Taken together, these results suggest that Ganweikang tablet intervention improves liver damage in MCD-induced NASH mice.

### Ganweikang Tablet Alleviates Hepatic Steatosis in MCD-Induced NASH Mice

**FIGURE 4:**
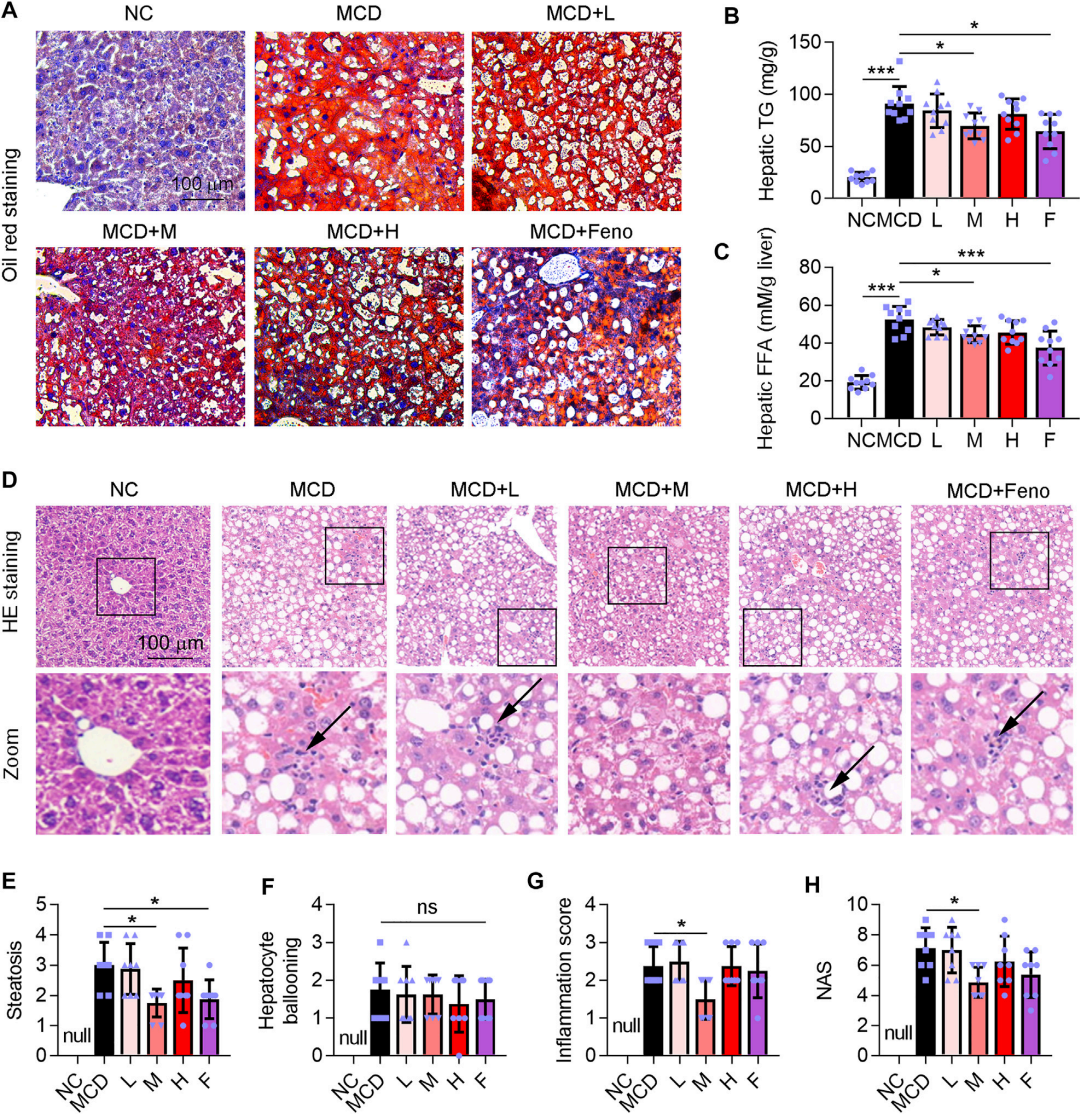
Ganweikang tablet alleviates steatosis in the liver of MCD-fed mice. **(A)** Representative images of oil red O staining on the liver sections. **(B,C)** Hepatic TG and FFA in different groups were detected by the kit. **(D)** Representative images of HE staining on the liver sections from the indicated groups. **(E–H)** After analysis of HE staining, the changes in steatosis **(E)**, hepatocyte ballooning **(F)**, inflammation score **(G)**, and NAS **(H)** between the groups are shown in the figure. *p<0.05, ***p<0.001, by one-way ANOVA with Bonferroni correction. ns, no significance.

Hepatic steatosis is a characteristic of NASH (Manne et al., 2018). Methionine and choline are essential precursors for lecithin biosynthesis in hepatocytes and are important substrates for VLDL synthesis and secretion. When the mice are treated with an MCD diet, the synthesis and secretion of VLDL are impaired, so that endogenous TG cannot be transported out of hepatocytes and is deposited in the liver, leading to fatty degeneration of hepatocytes (Rinella and Green, 2004). In this study, we observed a large accumulation of lipid droplets in the liver of the MCD-induced NASH mice model (Figure 4A), accompanied by a significant increase in TG and FFA levels in the liver, which was attenuated by the administration of Ganweikang tablet or fenofibrate (Figures 4A–C). Moreover, HE staining showed that hepatocyte ballooning was also significantly elevated in the mice under the condition of the MCD diet (Figure 4D). On the other hand, the MCD diet leads to a decrease in the precursors of antioxidants, also known as reactive methyl groups, followed by the activation of oxidative stress in the liver, which causes inflammation and apoptosis of hepatocytes (Merry et al., 2016). Accordingly, the HE staining showed that hepatic inflammation was also increased in the MCD-fed mice (Figure 4G). Intriguingly, similar to the fenofibrate group, these features of NASH, such as high hepatic TG and FFA level, hepatic steatosis, inflammation score, and NAS, were significantly decreased in the medium-dose Ganweikang tablet administration group, while hepatocyte ballooning was slightly reduced but without significance in the medium-dose Ganweikang tablet administration group (Figures 4B–H). The aforementioned results indicate that the Ganweikang tablet improves lipid deposition in the liver of MCD-induced NASH mice.

### Ganweikang Tablet Ameliorates Hepatic Inflammation and Fibrosis in MCD-Fed NASH Mice

**FIGURE 5:**
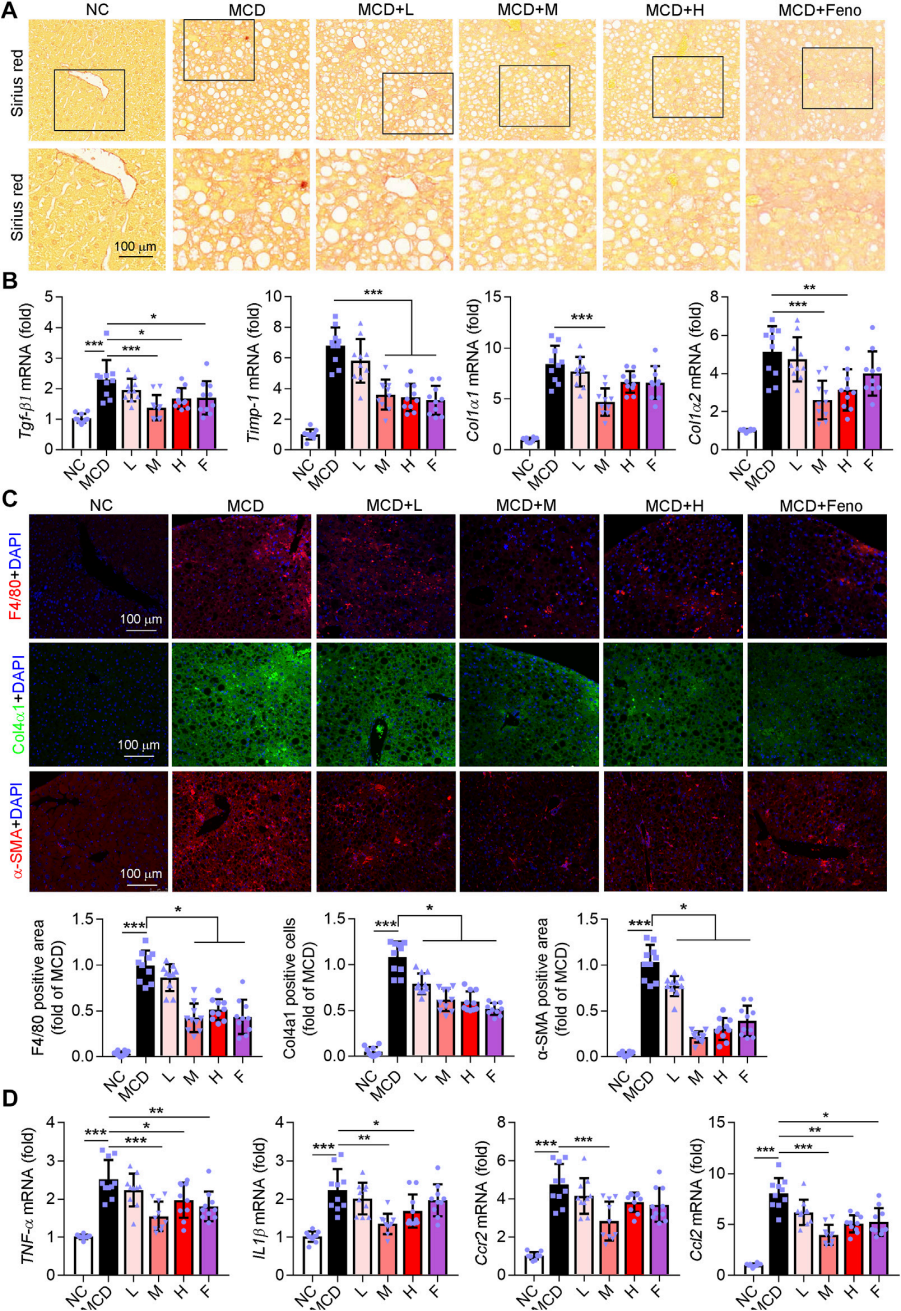
Ganweikang tablet ameliorates fibrosis and inflammation in the liver of MCD-fed NASH mice. **(A)** Representative images of Sirius Red staining, *n* = 10. **(B)** q-RT-PCR analysis of the mRNA level of *TNFα*, *IL1β*, *Ccr2*, and *Ccl2* in the liver, *n* = 10. **(C)** Mouse liver sections were stained for F4/80, Col4α1, and α-SMA, respectively, and followed by quantitative analysis, *n* = 10. F4/80 (red), Col4α1 (green), α-SMA (red), and DAPI (blue). **(D)** q-RT-PCR analysis of the mRNA level of Tgf-β1, Timp-1, Col1α1, and Col1α2 in the liver, *n* = 10. *p<0.05, **p<0.01, ***p<0.001, by one-way ANOVA with Bonferroni correction.

Fibrosis is accompanied by NASH development. Therefore, we determined whether the Ganweikang tablet could attenuate fibrosis in the NASH mice. Intriguingly, similar to the fenofibrate group, the data of Sirius Red staining showed that the Ganweikang tablet attenuated the fibrosis (Figure 5A). In addition, a medium dose of Ganweikang tablet significantly reduced the increase in liver fibrosis indicators, including *TGF-β1*, *Timp-1*, *Col1α1,* and *Col1α2* in NASH mice (Figure 5B). Fenofibrate reduced the expression of *TGF-β1* and *Timp-1* but did not affect the *Col1α1* and *Col1α2* expression in NASH mice (Figure 5B). Moreover, immunofluorescent staining on the fibrosis marker, such as Col4α1 and αSMA, was markedly decreased by fenofibrate and Ganweikang tablet treatment (Figure 5C). These data suggest that the Ganweikang tablet can reduce liver fibrosis in NASH mice. Hepatic macrophages act as a key regulator of liver fibrosis, and excessive accumulation of macrophages markedly contributes to liver fibrosis development (Krenkel et al., 2018). According to the F4/80 (macrophage marker) immunofluorescence staining, macrophage infiltration was significantly increased in MCD-fed mice compared to NC-fed mice, which was significantly attenuated in the fenofibrate group and the medium-dose Ganweikang tablet group (Figure 5C). Consistent with this, the mRNA levels of inflammatory factors, such as *TNFα*, IL1β, *Ccr2,* and *Ccl2*, in the liver were significantly reduced in the medium-dose Ganweikang-treated group (Figure 5D)*.* The aforementioned results suggest that the Ganweikang tablet can ameliorate inflammation and fibrosis in the liver of MCD-fed NASH mice.

### Network Pharmacology Analysis Indicates Ganweikang Tablet as a Candidate for NAFLD Treatment

**FIGURE 6:**
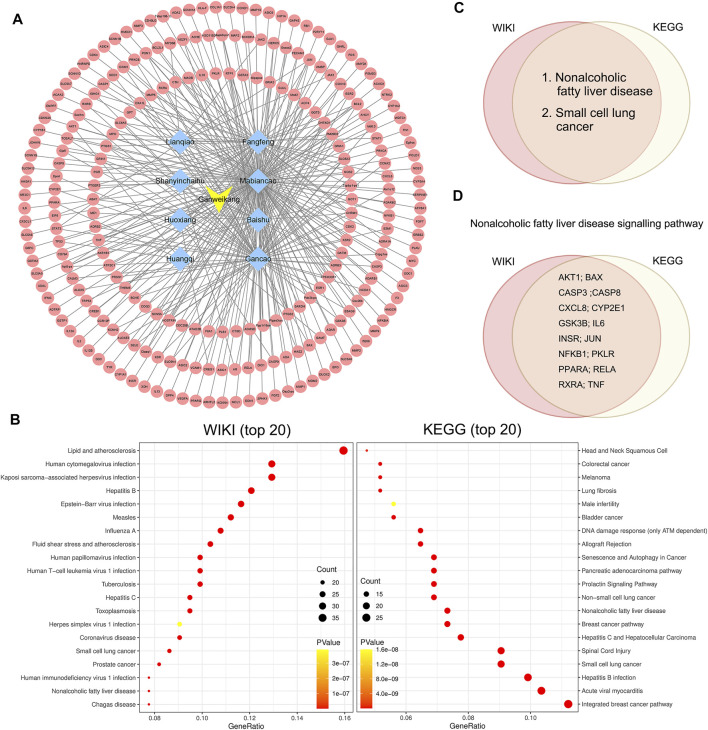
Network pharmacology analysis on target genes and disease of the Ganweikang tablet. **(A)** Ganweikang tablet (yellow)–Chinese herbal medicine (blue)–target genes (red) network. **(B)** Enrichment analysis of Ganweikang tablet target genes in WIKI and KEGG diseases (top 20). **(C)** Venn diagram representing the intersection of Ganweikang tablet target genes in KEGG and WIKI-enriched diseases. **(D)** Venn diagram representing Ganweikang target genes that are co-owned by KEGG and WIKI in NAFLD.

Furthermore, we conducted the network pharmacology analysis to determine the potential effect and molecular mechanism of Ganweikang tablet on the NAFLD. First, Ganweikang’s target genes were predicted from the HERB high-throughput database (http://herb.ac.cn/) based on literature data. Ganweikang tablet consists of eight Chinese herbal ingredients, including Lianqiao, Fangfeng, Shanyinchaihu, Mabiancao, Huoxiang, Baishu, Huangqi, and Gancao, which were individually predicted by HERB, resulting in the integration of 232 potential target genes for Ganweikang tablet (Figure 6A). In addition, these 232 genes were analyzed for enrichment by ERICHR (https://maayanlab.cloud/Enrichr/) and showcased the top 20 disease-related signaling pathways in the KEGG and WIKI databases (Figure 6B). In addition, we crossed the most significant gene enrichment in KEGG and WIKI databases and found that the targets of the Ganweikang tablet were mainly associated with NAFLD (Figure 6C). Furthermore, the predicted genes enriched in NAFLD in KEGG and WIKI databases were taken for intersection, and 16 genes were overlapped in KEGG and WIKI databases, including *AKT1*, *BAX*, *CASP3*, *CASP8*, *CXCL8*, *CYP2E1*, *GSK3B*, *IL6*, *INSR*, *JUN*, *NFκB1*, *PKLR*, *PPARα*, *RELA*, *RXRα*, and *TNF*α (Figure 6D). Taken together, network pharmacology analysis indicates that the Ganweikang tablet can serve as a candidate for NAFLD treatment and predicts the potential molecular mechanism underlying the action.

### Ganweikang Tablet Attenuates the NAFL and NASH by Inhibiting Inflammation, Apoptosis and Enhancing Fatty Acid Oxidation Through Inhibiting NFκB and caspase8 and Activating PPARα

**FIGURE 7:**
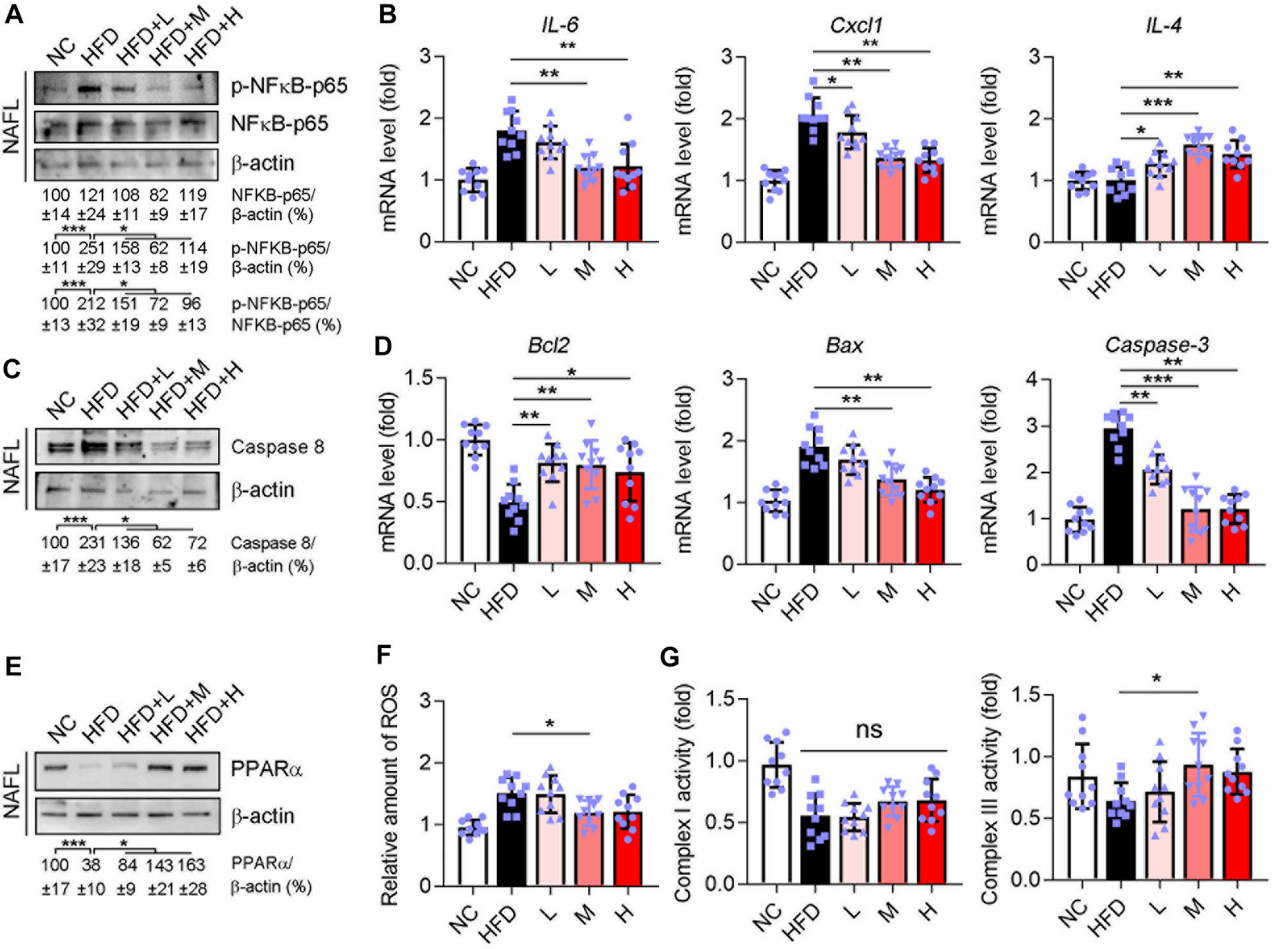
Ganweikang tablet reduces inflammation, apoptosis, and fatty acid oxidation in the liver of NAFL mice. C57BL/6J-background mice induced into NAFL model by HFD, *n* = 3. **(A)** Western blotting demonstrates the expression of p-NFκB-p65 and NFκB-p65, *n* = 3. **(B)** Differences in mRNA fold level changes of *IL6*, *Cxcl1,* and *IL4* between the indicated groups (*n* = 10 mice examined per group). **(C)** Western blotting demonstrates the expression of Caspase8, *n* = 3. **(D)** Differences in mRNA fold level changes of *Bcl2*, *Bax,* and *Caspase3* between the indicated groups (*n* = 10 mice examined per group). **(E)** Western blotting demonstrates the expression of PPARα, *n* = 3. **(F)** Differences in the amount of ROS between the indicated groups (*n* = 10 mice examined per group). **(G)** Differences in activity between the groups for complex I and complex III (*n* = 10 mice examined per group). *p<0.05, **p<0.01, ***p<0.001, by one-way ANOVA with Bonferroni correction. ns, no significance.

According to the network pharmacology analysis, the targeting genes of the Ganweikang tablet associated with NAFLD were mainly enriched in inflammation, apoptosis, and fatty acid oxidation. Therefore, further validation on the key targets of these signaling pathways was carried out in the liver of NAFL and NASH mice. Western blot results suggested that Ganweikang tablet significantly reduced the phosphorylation level of NF-κB in either NAFL or NASH liver (Figure 7A and Supplementary Figure S1A). Consistent with this result, the expression of the inflammatory factors *IL-6* and *Cxcl1* was significantly reduced after Ganweikang tablet treatment, while the expression of *IL-4* (anti-inflammatory cytokine) was significantly increased in either NAFL or NASH liver (Figure 7B and Supplementary Figure S1B). In the liver, caspase8, a protein that upregulates apoptosis, was reduced by intervention with the Ganweikang tablet (Figure 7C and Supplementary Figure S1C). In addition, Ganweikang tablet significantly inhibited the mRNA levels of *Bax* and *Caspase3* in NAFL and NASH mice and increased *Bcl2* mRNA level (Figure 7D and Supplementary Figure S1D). PPARα is an important target for the upregulation of fatty acid oxidation (FAO). Inhibition of PPARα in NAFLD leads to reduced FAO and mitochondrial dysfunction (Pawlak et al., 2015). In this study, the expression of PPARα was significantly reduced in NAFL and NASH mice compared to the NC group; however, Ganweikang tablet partially reversed this effect (Figure 7E and Supplementary Figure S1E), suggesting that Ganweikang tablet could increase FAO levels in the liver of NAFLD model mice. When the hepatocyte was exposed to excess FFAs for a prolonged time, oxidative stress can be activated, prompting the release of large amounts of reactive oxygen species (ROS) from the mitochondria. In line with the level of PPARα expression, the medium-dose Ganweikang tablet reduced hepatic ROS (Figure 7F and Supplementary Figure S1F), further implying that Ganweikang tablets can ameliorate oxidative stress. Mitochondria are a source of ROS and a major organelle for ROS attack. Herein, we explored the state of mitochondrial biological function by assaying the activity of mitochondrial complexes I and III. We found that Ganweikang tablets could increase the activity of mitochondrial complex III in NAFL and NASH mice (Figure 7G and Supplementary Figure S1G), which maintained normal mitochondrial function. The aforementioned results suggest that the Ganweikang tablet can reduce inflammation and apoptosis and enhance FAO by inhibiting NFκB and Caspase8 and activating PPARα, which protected the mice from NAFL and NASH suffering.

### Study on the Molecular Mechanism of Ganweikang Tablet Improving NAFL and NASH *via* Network Pharmacology Combined Cell Experiment

**FIGURE 8:**
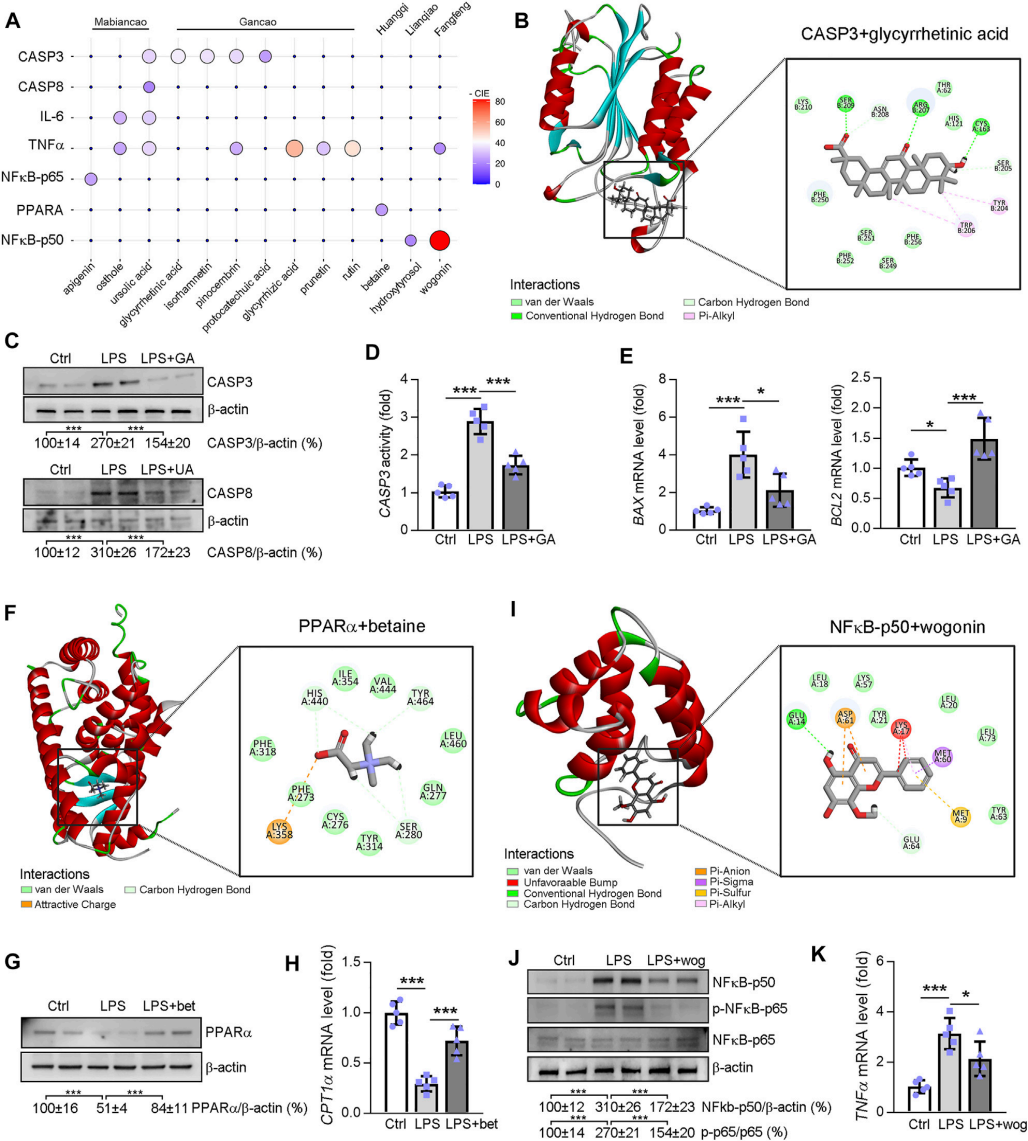
Molecular docking between the potential molecule targets and the compound analyzed by network pharmacology. **(A)** The ggballoon plot demonstrates the degree of interaction between Ganweikang’s potent small molecule compounds and the receptor proteins. The strength of the -CIE is shown by the size and color of the circles. 0 means that the relationship between the active ingredients and the proteins has not been predicted by the HERB database (http://herb.ac.cn/). **(B)** Molecular docking of glycyrrhetinic acid and CASP3. **(C)** Western blotting demonstrates the protein level of CASP3 and CASP8 in HepG2 cells after indicated treatment, *n* = 3. **(D)** Casp3 activity in HepG2 cells was determined by the commercial kit, *n* = 5. **(E)** Transcriptional expression of BAX and BCL2 in HepG2 cells after indicated treatment was determined by qRT-PCR, *n* = 5. **(F)** Molecular docking simulations of betaine and PPARα. **(G,H)** After indicated treatment, expression of PPARα and CPT1α in HepG2 cells was determined by Western blotting and qRT-PCR, *n* = 5. **(I)** Molecular docking simulations of NFκB-p50 and wogonin. **(J,K)** After indicated treatment, expression of NF-κB-p50, NF-κB-p65, p-NF-κB-p65, and TNFα in HepG2 cells was determined by Western blotting and qRT-PCR, n = 5. *p<0.05, ***p<0.001, by one-way ANOVA with Bonferroni correction.

Visualization of -Cdocker interaction energy (CIE) between the Ganweikang tablet’s active ingredients that were analyzed by network pharmacology and the corresponding target genes that we have determined *in vivo* experiment is shown in Figure 8A. First, molecular docking simulations of the binding sites and the interaction forces of glycyrrhetinic acid (GA), CASP3 (Figure 8B), ursolic acid (UA), and CASP8 (Supplementary Figure S2A) were performed. Subsequently, Western blot analysis showed that LPS (1 μg/ml) induced an increase in protein levels of CASP3 and CASP8 in HepG2 cells, which was reversed by GA (20 μg/ml) and UA (5 μM) treatment, respectively (Figure 8C). In addition, CASP3 activity was enhanced by LPS, while GA treatment inhibited CASP3 activity (Figure 8D). Moreover, the mRNA expression levels of *BAX* and *BCL2* were increased and decreased by LPS, respectively, and GA significantly attenuated the effect of LPS on these *BAX* and *BCL2* expressions (Figure 8E). Next, molecular docking simulations of betaine and PPARα are shown in Figure 8F. In addition, the PPARα protein level was reduced by LPS while betaine (5.0 mg/ml) restored its expression (Figure 8G). The mRNA level of CPT1α (the downstream gene of PPARα) supported the finding that betaine treatment increased the activity of PPARα in the presence of LPS (Figure 8H). Molecular docking simulations of NFκB-p50 and wogonin are exhibited in Figure 8I. Furthermore, LPS increased NFκB-p50 expression in HepG2 cells, which was inhibited by wogonin (10 μg/ml) (Figure 8J). In addition, wogonin inhibited the enhanced NFκB-p65 phosphorylation caused by LPS (Figure 8J). Moreover, *TNFα* mRNA expression levels were increased by LPS and wogonin treatment significantly inhibited this change (Figure 8K). Moreover, the molecular docking prediction of GA and TNFα, UA and IL6, NFκB, and apigenin are presented in Supplementary Figure S2. Taken together, the aforementioned results confirmed that the active ingredient of Ganweikang that was analyzed by network pharmacology can ameliorate LPS-induced changes in apoptosis, fatty acid oxidation, and inflammatory signaling pathways, possibly by which Ganweikang tablet protects the liver from NAFL and NASH in the mice.

## Discussion

NAFLD, a liver disease associated with obesity, insulin resistance, type 2 diabetes mellitus, hypertension, hyperlipidemia, and metabolic syndrome, is now considered a significant driver of the global burden of chronic liver disease and can carry serious sequelae (Golabi et al., 2021). There is a potential progression of NAFLD from simple steatosis (NAFL) to more severe NASH, with terminal progression to liver fibrosis, cirrhosis, and hepatocellular carcinoma, which places a heavy financial and life burden on patients (Younossi, 2019). However, to date, there are no FDA-approved drugs for the treatment of NAFLD. The pathogenesis of NAFLD is complex and no single-target drugs have achieved the desired results. Noticeably, traditional Chinese medicine has gained increasing attention for NAFLD treatment due to its multi-target and multi-pathway advantages. The underlying mechanism of action of Ganweikang tablet, a compounded traditional Chinese medicine, is not yet clear. Fortunately, network pharmacology proposed by Hopkins University in 2007 is a comprehensive discipline that integrates systems biology, information networks, computer science, and pharmacology (20), which can provide a theoretical basis for predicting the target genes and diseases potentially treated by compounded herbal medicines. In this study, we found that NAFLD is a major target disease of Ganweikang’s major components, with target genes associated with inflammation, apoptosis, and fatty acid oxidation, which provide a theoretical basis for its treatment of NAFLD. Accordingly, we investigated the therapeutic effects of Ganweikang tablet on two stages of NAFLD pathogenesis through different disease models, NAFL and NASH mice, respectively. The results suggested that the Ganweikang tablet significantly reduced lipid deposition, fibrosis, and inflammation in the liver from both the NALF and NASH models.

Hepatic lipid accumulation, inflammation, and apoptosis are major contributors to NAFLD development (Friedman et al., 2018). In this study, lipid accumulation, macrophage infiltration, and collagen deposition were significantly reduced in NAFL and NASH mice after Ganweikang tablet intervention. Kupffer cells and newly recruited monocyte-derived macrophages play key roles in the regulation of inflammation, fibrosis, and fibrinolysis. Following injury, Kupfer cells recruit inflammatory blood monocytes, which differentiate into classically activated M1-type macrophages with the secretion of large amounts of pro-inflammatory cytokines and ROS (Kazankov et al., 2019). In contrast, M2-type macrophages with anti-inflammatory and repair phenotypes are associated with reduced liver injury in NAFLD (Wan et al., 2014). Our study indicates that the Ganweikang tablet can significantly promote the M2 subtypes of macrophages and reduce pro-inflammatory cytokines in the liver of mice in the NAFL model. The flow of monocytes to the liver is mainly regulated by the chemokine CCL2 and its cognate receptor CCR2 in monocytes or macrophages (Obstfeld et al., 2010). In this study, under the treatment of Ganweikang tablets, the levels of *Ccl2* and *Ccr2* in the liver of the NAFL mice were reduced, accompanied by a decrease in the expression of the pro-inflammatory factor TNF-α, which accounts for decreased macrophages infiltration and reducing inflammation in the liver of NAFL and NASH mice. In addition, fibrosis-related indicators in the liver were significantly reduced, especially in the medium-dose Ganweikang group, suggesting a protective effect on hepatic fibrosis in the NASH model.

Mechanistically, network pharmacology analysis showed that the regulated genes of Ganweikang tablet mainly enriched in the signaling pathway of fatty acid metabolism (PPARα), inflammation response (NFκB-p65), and apoptosis (Casp3 and Casp8). *PPARα* is a key gene in the regulation of fatty acid metabolism, which can affect the hepatic lipid level (Chung et al., 2018). Huangqi, as the main component of the Ganweikang tablet, can affect *PPARα* activity (Cao et al., 2021). In this study, the Ganweikang tablet increased the expression of PPARα, suggesting that the Ganweikang tablet may increase the hepatic FAO and thereby attenuating NAFL and NASH. In addition, network pharmacology predicts that *PPARα* is the target gene of betaine (the active ingredient of Huangqi). Furthermore, betaine treatment increased the protein level of PPARα and its regulated gene expression, CPT1α, in HepG2 cells in the presence of LPS. In addition, NFκB is an important factor in regulating the inflammatory signaling pathway (Cartwright and Perkins, 2016). Network pharmacology analysis indicated that NFκB is a potential target gene of Lianqiao, a component of the Ganweikang tablet. Indeed, after treatment with a Ganweikang tablet, the activity of NFκB was markedly decreased in the liver of NAFL and NASH mice. In line with the inactivation of NFκB, the inflammatory cytokines were also decreased after Ganweikang tablet treatment, which suggested that the treatment of NAFLD by Ganweikang tablet is partially through inactivating NFκB. Furthermore, network pharmacology analysis indicated that NFκB is a potential target gene of wogonin (the active ingredient of Fangfeng from the Ganweikang tablet). *In vivo*, after treatment with a Ganweikang tablet, the phosphorylation of NFκB-p65 was markedly decreased in the liver of NAFL and NASH mice. *In vitro*, wogonin treatment inhibited the expression of NFκB-p50 and phosphorylation of NFκB-p65 in HepG2 cells in the presence of LPS. Additionally, the mRNA level of *TNFα* also supported our results that wogonin decreased LPS-induced inflammation in HepG2 cells. In addition, *CASP3* and *CASP8* are apoptosis-associated key genes (Mata et al., 2010). The network pharmacology approach predicts that *CASP3* and *CASP8* are the target genes of UA and GA, two components of that Gancao and Mabiancao that are contained in the Ganweikang tablet. As expected, the markers of apoptosis were significantly reduced in the liver of NAFL and NASH mice after Ganweikang tablet treatment. In line with this, the *in vitro* experiment showed that GA treatment inhibited LPS-induced markers of apoptosis in HepG2 cells. These results showed that betaine-mediated PPARα activation, GA-mediated inactivation of CASP3, UA-mediated downregulation of CASP8, and wogonin-mediated inactivation of NFκB may be the potential mechanisms of Ganweikang tablet in the treatment of NAFLD. Altogether, Ganweikang tablet attenuated the NAFLD development by attenuating the hepatic lipid accumulation, inflammation, and apoptosis through the different herbal components, suggesting that Ganweikang tablet can ameliorate the disease by a multi-target effect. Moreover, *in vivo* experiments revealed that medium-dose Ganweikang tablet (672 mg/kg/day) significantly improved liver injury in NAFLD mice, in some respects, which showed a better effect than that of low- and high-dose treatments. Commonly, the lower dose of the drug was safer than the high dose in the treatment of NAFLD. Meanwhile, given the comparable effect between medium- and high-dose Ganweikang tablets, we suggested that the medium dose of Ganweikang tablet was better. Therefore, the optimal dose for Ganweikang tablet administration was considered at 672 mg/kg/day in this study.

## Conclusion

In summary, we have observed that Ganweikang tablet can improve liver lesions in a mouse model of NAFL and NASH by modulating inflammation, apoptosis, and fatty acid oxidation through inhibiting NFκB, inactivating caspase3/8, and activating PPARα, which indicates Ganweikang tablet not only as a drug candidate but also provides a theoretical basis of Ganweikang tablet in the treatment of NAFLD.

## Data Availability

The original contributions presented in the study are included in the article/Supplementary Material; further inquiries can be directed to the corresponding authors.

## Abbreviations

ALT, alanine transaminase; ALP, alkaline phosphatase; AST, aspartate transaminase; CCR-2, C–C chemokine receptor type-2; CASP8, caspase8; IL-1β, interleukin 1 beta; NF-κB, nuclear factor κB; and PPARα, peroxisome proliferator-activated receptor-α.
