# Supplementary material for: Exploring Ganweikang Tablet as a Candidate Drug for NAFLD Through Network Pharmacology Analysis and Experimental Validation
Source: Front Pharmacol. 2022 Jun 14;13:893336. doi: 10.3389/fphar.2022.893336 (PMC9239345; doi:10.3389/fphar.2022.893336)
Supplement: Supplementary file 1 [file DataSheet1.docx]

**Supplementary materials**

**Exploring GanWeiKang tablet as a candidate drug for NAFLD through network pharmacology analysis and experimental validation**

**
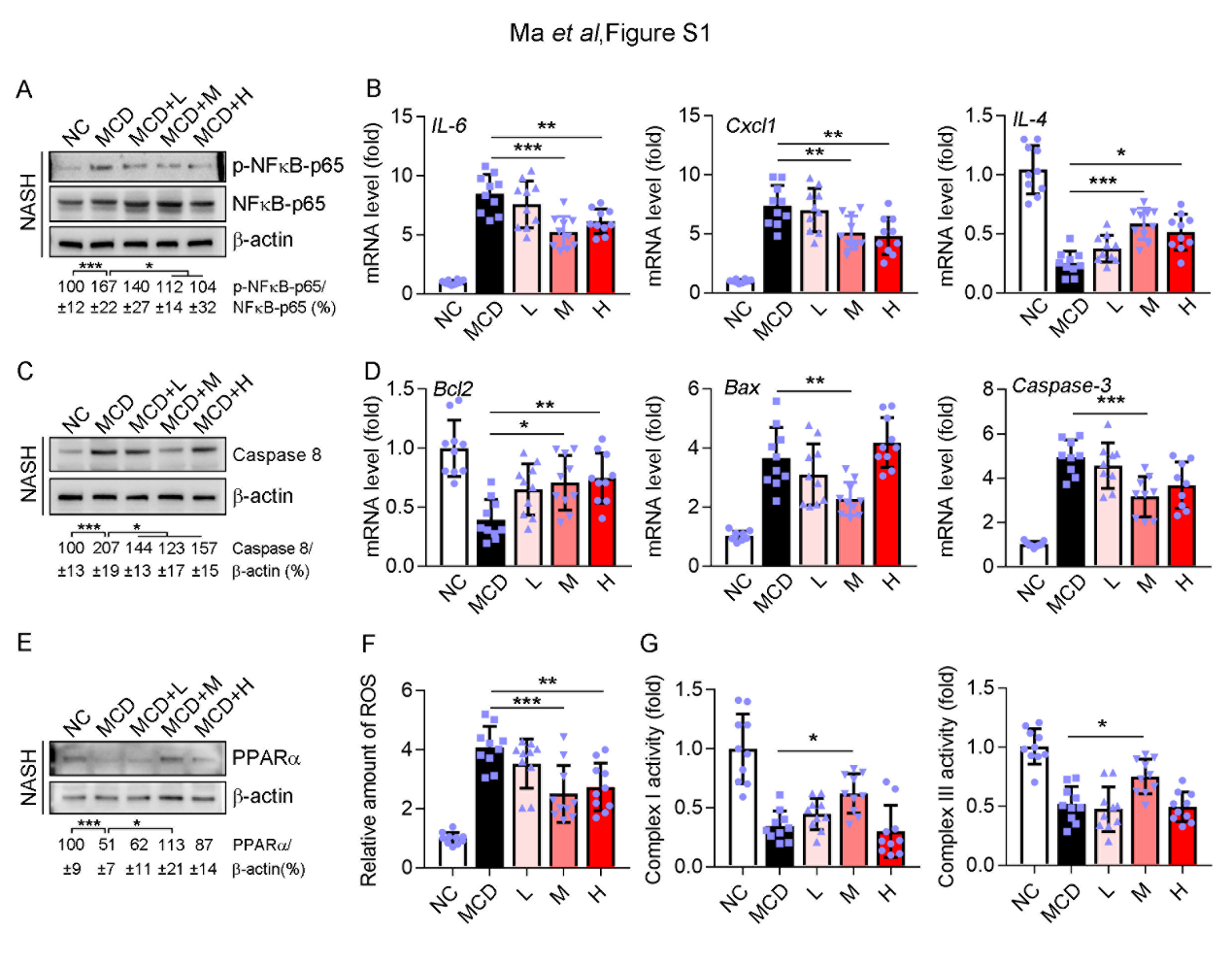
**

**Figure S1. Ganweikang reduces expression of genes responsible for inflammation, apoptosis and promotes expression of gene responsible for fatty acid oxidation in the liver of NASH mice.** C57 BL/6J-background mice induced into NAFL model by MCD. **(A)** Western blotting demonstrating the expression of p-NFκB-p65 and NFκB-p65, n=3. **(B)** Differences in mRNA fold level changes of *IL6*, *Cxcl1* and *IL4* between the groups (n = 10 mice examined per group). **(C)** Western blotting demonstrating the expression of Caspase8, n=3. **(D)** Differences in mRNA fold level changes of *Bcl2*, *Bax* and *Caspase3* between the groups (n = 10 mice examined per group). **(E)** Western blotting demonstrating the expression of PPARα, n=3. **(F)** Differences in the amount of ROS between the groups (n = 10 mice examined per group). **(G)** Differences in activity between the groups for complex 1 and complex 3. (n = 10 mice examined per group). **p*＜0.05, ***p*＜0.01, ****p*＜0.001, by one-way ANOVA with Bonferroni correction.

**
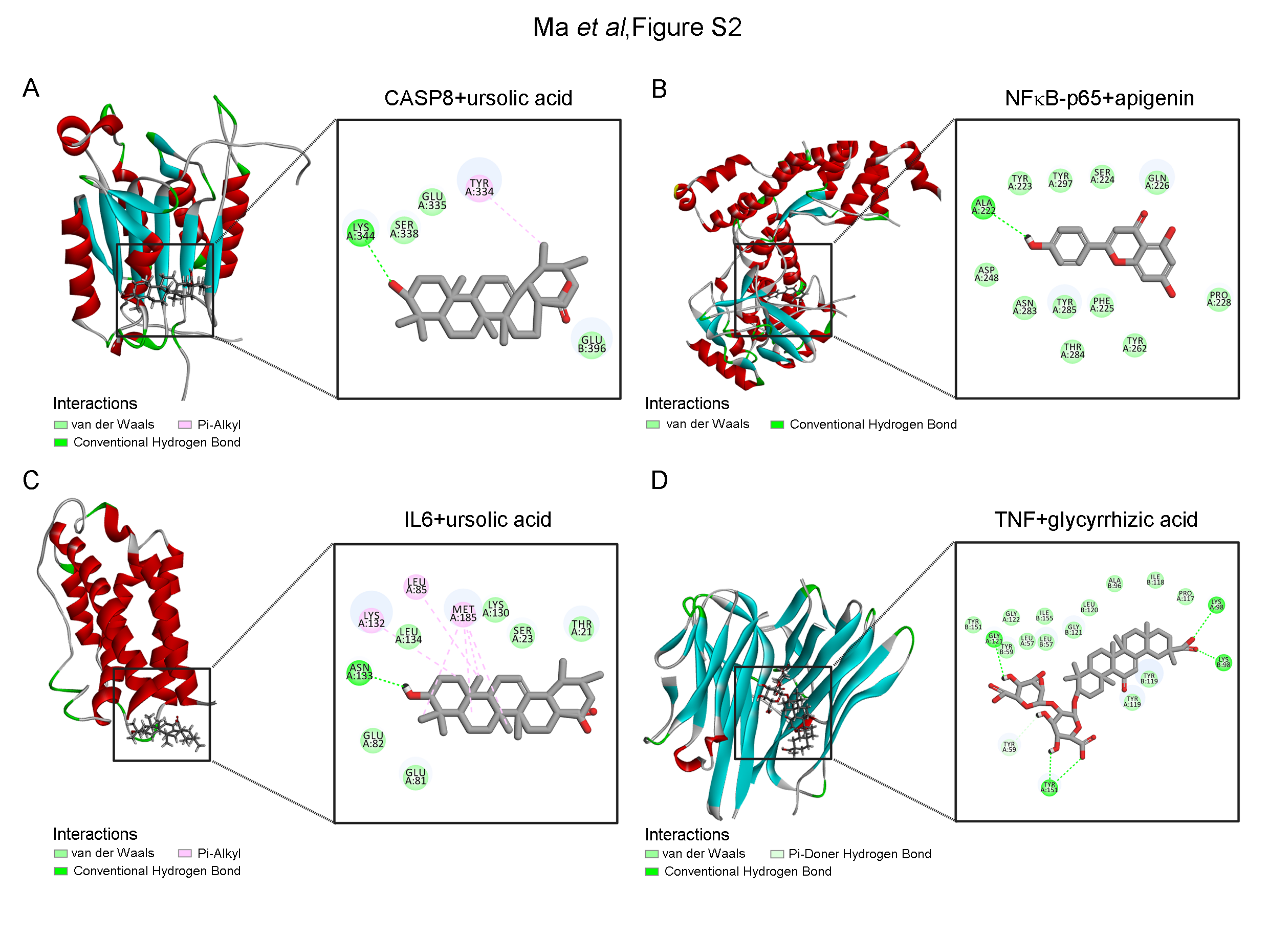
**

**Figure S2. Molecular docking between the potential molecule targets and the predicted compound that** **analyzed by Network pharmacology.** (**A**) Molecular docking prediction of ursolic acid and CASP8. (**B**) Molecular docking prediction of apigenin and NF-Kb-p65. (**C**) Molecular docking prediction of ursolic acid and IL6. (**C**) Molecular docking prediction of glycyrrhetinic acid and TNFα.

**Table S1. Sequences of primers for q-RT-PCR**

| Gene | Forward | Backward |
| --- | --- | --- |
| *mTNF-α(ID:21926)* | ***GACGTGGAACTGGCAGAAGAG*** | ***TTGGTGGTTTGTGAGTGTGAG*** |
| *mCcr2(ID:12772)* | ***ATCCACGGCATACTATCAACATC*** | ***CAAGGCTCACCATCATCGTAG*** |
| *mCcl2(ID:20296)* | ***TTAAAAACCTGGATCGGAACCAA*** | ***GCATTAGCTTCAGATTTACGGGT*** |
| *mCol1a1(ID:12842)* | ***GCTCCTCTTAGGGGCCACT*** | ***CCACGTCTCACCATTGGGG*** |
| *mTimp1(ID:21857)* | ***GCAACTCGGACCTGGTCATAA*** | ***CGGCCCGTGATGAGAAACT*** |
| *mMmp9(ID:17395)* | ***CTGGACAGCCAGACACTAAAG*** | ***CTCGCGGCAAGTCTTCAGAG*** |
| *mIL-6(ID:* *16193)* | ***TAGTCCTTCCTACCCCAATTTCC*** | ***TTGGTCCTTAGCCACTCCTTC*** |
| *mCxcl1(ID:* *14825)* | ***CTGGGATTCACCTCAAGAACATC*** | ***CAGGGTCAAGGCAAGCCTC*** |
| *mIL-4(ID:* *16189)* | ***GGTCTCAACCCCCAGCTAGT*** | ***GCCGATGATCTCTCTCAAGTGAT*** |
| *mBcl2(ID:* *12043)* | ***GTCGCTACCGTCGTGACTTC*** | ***CAGACATGCACCTACCCAGC*** |
| *mBax(ID:* *12028)* | ***TGAAGACAGGGGCCTTTTTG*** | ***AATTCGCCGGAGACACTCG*** |
| *mCol1a2(ID:* *12843)* | ***GTAACTTCGTGCCTAGCAACA*** | ***CCTTTGTCAGAATACTGAGCAGC*** |
| *mCasp3 (ID:* *12367)* | ***ATGGAGAACAACAAAACCTCAGT*** | ***TTGCTCCCATGTATGGTCTTTAC*** |
| *mIL-1β(ID:16176)* | ***GCAACTGTTCCTGAACTCAACT*** | ***ATCTTTTGGGGTCCGTCAACT*** |
| *mTgfβ1(ID:21803)* | ***CTCCCGTGGCTTCTAGTGC*** | ***GCCTTAGTTTGGACAGGATCTG*** |
| *β-actin (ID: 11461)* | ***GGCTGTATTCCCCTCCATCG*** | ***CCAGTTGGTAACAATGCCATGT*** |
